# Similarities between acylcarnitine profiles in large for gestational age newborns and obesity

**DOI:** 10.1038/s41598-017-15809-4

**Published:** 2017-11-24

**Authors:** Paula Sánchez-Pintos, Maria-Jose de Castro, Iria Roca, Segundo Rite, Miguel López, Maria-Luz Couce

**Affiliations:** 1 Diagnosis and Treatment of Congenital Metabolic Diseases Unit (UDyTEMC). Neonatology Service. Department of Pediatrics. Hospital Clínico Universitario. University of Santiago de Compostela. Institute of Clinical Research of Santiago de Compostela (IDIS). CIBERER, Santiago de Compostela, Spain; 2 Neonatology Unit. University Hospital Miguel Servet, Zaragoza, Spain; 3 NeurObesity Group. Department of Physiology, CIMUS. University of Santiago de Compostela. Institute of Clinical Research of Santiago de Compostela (IDIS), Santiago de Compostela, 15782 Spain; 4 CIBER Fisiopatología de la Obesidad y Nutrición (CIBERobn), Santiago de Compostela (IDIS), 15706 Spain

## Abstract

Large for gestational age (LGA) newborns have an increased risk of obesity, insulin resistance, and metabolic syndrome. Acylcarnitine profiles in obese children and adults are characterized by increased levels of C3, C5, and certain medium-chain (C12) and long-chain (C14:1 and C16) acylcarnitines. C2 is also increased in insulin-resistant states. In this 1-year observational study of 2514 newborns (246 LGA newborns, 250 small for gestational age (GA) newborns, and 2018 appropriate for GA newborns), we analyzed and compared postnatal acylcarnitine profiles in LGA newborns with profiles described for obese individuals. Acylcarnitine analysis was performed by tandem mass spectrometry on dried-blood spots collected on day 3 of life. LGA newborns had higher levels of total short-chain acylcarnitines (*p* < 0.001), C2 (*p* < 0.01) and C3 (*p* < 0.001) acylcarnitines, and all C12, C14, and C16 acylcarnitines except C12:1. They also had a higher tendency towards carnitine insufficiency (*p* < 0.05) and carnitine deficiency (*p* < 0.001). No significant differences were observed between LGA newborns born to mothers with or without a history of gestational diabetes. This novel study describes a postnatal acylcarnitine profile in LGA with higher levels of C2, C3, total acylcarnitines, and total short-chain acylcarnitines that is characteristic of childhood and adult obesity and linked to an unhealthy metabolic phenotype.

## Introduction

Large for gestational age (LGA) is defined as a birth weight above the 90^th^ percentile for the corresponding gestational age (GA)^1^. The maternal factors most closely associated with LGA are maternal obesity, excessive gestational weight gain, maternal gestational diabetes mellitus (GDM), pregestational obesity, and maternal stress^2–9^. Fetal factors associated with LGA consist primarily of genetic or chromosomal disorders. LGA prevalence is estimated at between 4.6% and 15.3%^10^ and is influenced by ethnicity, with higher rates found in children born to African American and non-Hispanic Asian American women in U.S. studies^11,12^.

Excessive fetal growth has negative consequences that extend beyond the neonatal period and these include medium- and long-term neurological, behavioral, and cardiovascular impacts^13–16^. LGA newborns are also at an increased risk of obesity^17–20^, metabolic syndrome^21,22^, and insulin resistance^23^ in later life. They have also been found to have elevated leptin and fasting insulin and homeostasis model assessment (HOMA) index levels^24^ during childhood, in addition to elevated adiponectin levels^25^, despite previous reports to the contrary from other studies of insulin states. The risk of obesity in LGA newborns increases with co-occurrence of maternal overweight/obesity or diabetes mellitus^26^.

Dysregulation of fatty acid oxidation and subsequent lipotoxicity play an important role in the pathophysiology of obesity-induced insulin resistance^27,28^. Analysis of acylcarnitine profiles by tandem mass spectrometry (MS/MS) in dried-blood spots has been used to investigate fatty acid oxidation alterations in obesity and type 1 and type 2 diabetes mellitus in both human and animal models^29–32^. A recent systematic review, however, failed to identify a consistent metabolite profile in GDM^33^.

The aim of this study was to characterize postnatal plasma acylcarnitine profiles in a cohort of LGA newborns. As a secondary outcome, we analyzed and compared the acylcarnitine fingerprint of LGA infants born to mothers with and without gestational diabetes (LGA-GDM and LGA-noGDM respectively).

## Results

### General characteristics of the study population

In total, 2514 newborns (1362 males and 1152 females) were included over the 1-year study period. None of them met the exclusion criteria. There were 2302 full-term newborns and 212 preterm newborns, with a medium GA of 39 weeks and the following anthropometric characteristics: mean birth weight, 3225 ± 591 g; mean birth length, 49.05 ± 2.47 cm; and mean head circunference, 34.3 ± 1.66 cm. In the preterm group, the respective measurements were 2127 ± 644 g, 44.03 ± 3.88 cm, and 31.13 ± 2.42 cm. The distribution according to birth weight percentile was 9% for small for GA (SGA) newborns (250/2514), 80.2% for appropriate for GA (AGA) newborns (2018/2514), and 9.7% for LGA newborns (246/2514). All birth measurements were higher in LGA newborns: weight, 4118 ± 234 g; length, 51.95 ± 1.36 cm; and head circumference, 35.97 ± 1.36 cm. Only one preterm newborn included in the study was classified as LGA. A flow diagram of the cohort is shown in Supplementary Figure 1.

The percentage of newborns in the severe LGA group (>97^th^ percentile) was 3% (75/2514), which is identical to the percentage of newborns in the severe SGA group (<3^rd^ percentile).There were thus 2364 newborns in the AGA^(3-97)^ group, which contained newborns with a birth weight ≥3^rd^ percentile and ≤97^th^ percentile.

Nine percent (246/2514) of the newborns were born to mothers with a history of GDM; 81.4% of the mothers received dietary treatment and 18.6% required insulin treatment. The breakdown of the LGA group was as follows: 42 LGA-GDM newborns and 204 LGA-noGDM newborns. No significant differences were observed between the mothers in these subgroups for either age or obstetric comorbidities (Supplementary Table 1).

### Acylcarnitine profiles

**Figure 1:**
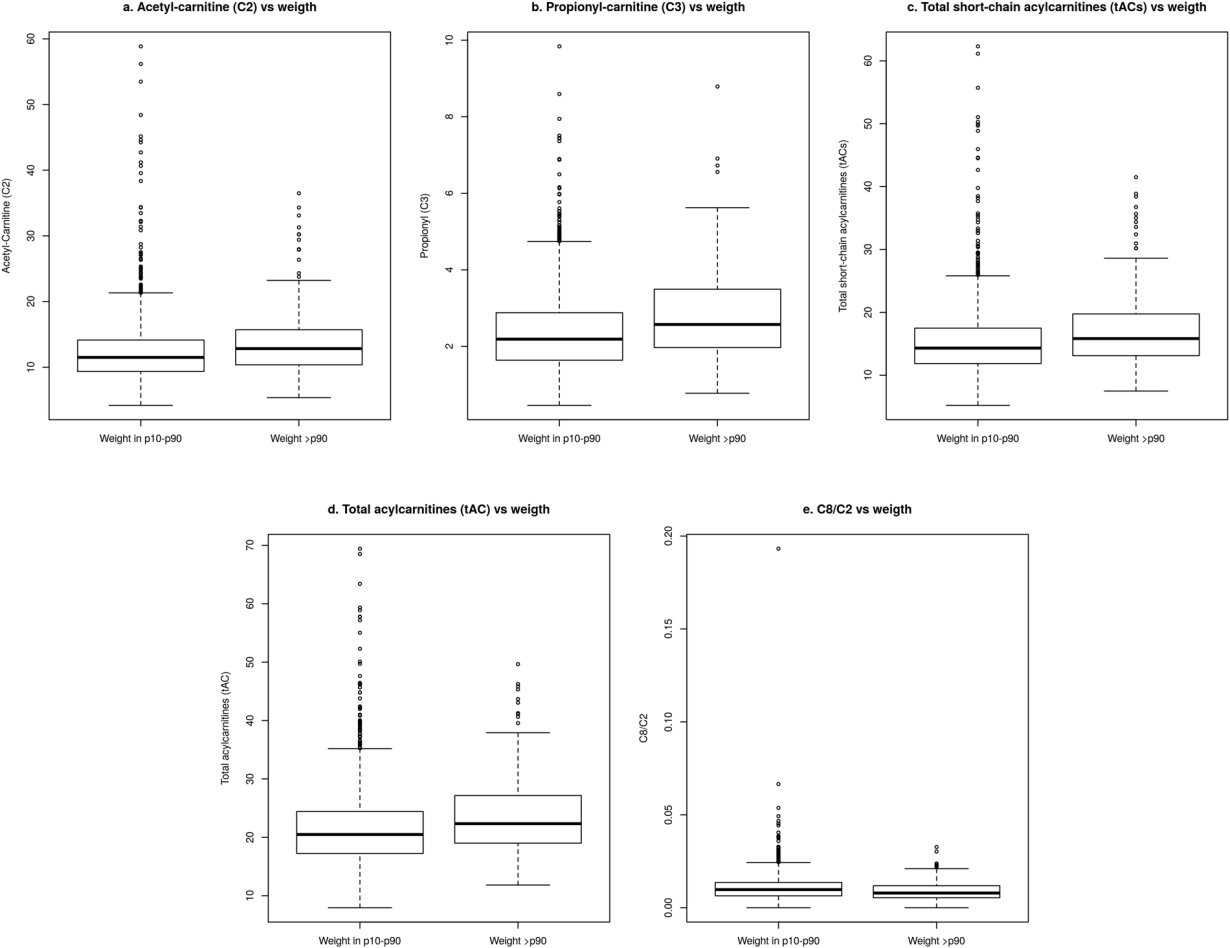
Main differences between large for gestational age (LGA) and appropriate for gestational age (AGA) newborns.

All acylcarnitine values are expressed as medians and the corresponding 95% confidence interval is given in the tables. Compared with AGA newborns, LGA newborns had higher levels of FC, TC, tAC (*p* < 0.01), tACm, tACl, and, in particular, tACs (*p* < 0.01). As shown in Table 1, LGA newborns had the highest tAC/FC ratio (0.795) and the lowest FC/TC ratio (0.557). This was close to the cutoff for neonatal carnitine insufficiency (tAC/FC > 0.83) and carnitine deficiency (FC/TC < 0.54), suggesting reduced carnitine storage in this group (SGA, 0.603; AGA, 0.578; and LGA, 0.557) (*p* < 0.001). Separate analysis of the various acylcarnitines showed remarkably higher levels of C2 and C3 in LGA newborns (*p* < 0.01), although plasma concentrations for the majority of short- and long-chain acylcarnitines were also higher in the LGA group. The C8/C2 ratio was considerably lower in the LGA group (0.0078 vs. 0.0097 for the AGA group, *p* < 0.01). The most significant differences between LGA and AGA newborns are summarized in Fig. 1

**Table 1:** Acylcarnitine profiles (µmol/L) according to birth weight based on the 10^th^ and 90^th^ percentiles.

	SGA (n: 250)		AGA (n:2018)		LGA (n:246)		SGA vs AGA		AGA vs LGA	
	Median	Range	Median	Range	Median	Range	*P* ^*1*^	*95% CI*	*P* ^*2*^	*95%CI*
**FC**	33.58	8.27–80.24	27.96	7.12–100.56	28.20	9.23–80.16	**6.8e** ^**−8**^	3.73, 6.72	*NS*	−0.90, 1.75
**TC**	57.57	17.00–118.44	49.01	20.05–135.89	51.20	23.26–114.70	**2.09e** ^**−7**^	5.68, 10.36	*NS*	0.34, 4.47
**tAC**	22.16	8.72–55.98	20.49	7.95–69.41	22.34	11.83–49.65	**0.0003**	1.17, 2.88	**0.0003**	1.11, 2.77
**tACs** **C2** **C3** **C3:1** **C4** **C4-OH** **C5** **C5:1** **C5-OH**	16.5813.332.760.0010.0020.2760.2510.0280.153	6.16–50.204.06–43.780.51–10.060–0.5530–1.6320–0.9110.03–1.0480–0.2630.007–0.43	14.2911.502.190.0090.0020.2600.1760.0260.151	5.19–62.234.19–58.850.46–9.830–1.750–1.6770–1.7470–1.5780–0.8060–0.888	15.8212.832.570.0070.2760.2700.1790.0260.148	7.47–41.435.35–36.480.77–8.780–0.4930–1.5260.045–1.9630.018–1.090–0.3020–0.713	**4.86e** ^**−7**^ **0.0002** **5.71e** ^**−10**^ *NS* **8.73e** ^**−4**^ *NS* **3.94e** ^**−16**^ *NS* *NS*	1.78, 3.21.05, 2.220.41, 0.70−4.0e^**−**5^, 1.5e^**−**5^0.038, 0.078−0.009, 0.0250.062, 0.095−5.9e^**−**5^, 0.003−0.005, 0.011	**0.0001** **0.007** **4.42e** ^**−7**^ *NS* *NS* *NS* *NS* *NS* *NS*	0.91, 2.230.66, 1.730.27, 0.54−2.9e^**−**5^, 5.2e^**−**5^−0.009, 0.012−0.012, 0.023−0.01, 0.011−0.001, 0.002−0.005, 0.011
**tACm** **C6** **C6**–**OH** **C8** **C8:1** **C10** **C10:1** **C10:2** **C5DC** **C4DC** **C12** **C12:1** **C12:2**	**0.770**0.0630.0610.1210.2170.1140.1060.0610.080.2800.1370.0830.027	0.171–2.610–0.6630–0.2440–0.3610–0.6750–0.4820–0.4140–0.2960–0.3320.04–0.8340–0.6280–0.380–0.153	**0.805**0.0400.0500.1100.1800.1280.1010.0520.0670.3320.1520.0930.026	0.109–4.980–0.7310–0.4790–2.3940–0.6830–0.9660–0.790–0.4140–0.3950–1.0630–1.7890–0.5020–0.277	**0.773**0.5240.0540.1110.2030.1200.0990.0500.0630.3340.1590.0900.025	0.202–7.0570–0.3490–0.4030–0.460–0.5810–0.9880–0.7530–0.3950–0.2960.11–1.1060.03–4.3910–0.4610–0.197	*NS* *NS* *NS* *NS* *NS* *NS* *NS* *NS* *NS* **1.06e** ^**−9**^ **0.002** *NS* *NS*	−0.021, 0.059.4e–6, 0.013−0.002, 0.008−0.002, 0.0140.012, 0.039−0.019, 0.002−0.006, 0.0110.002, 0.0150.003, 0.015−0.071, −0.039−0.029, −0.005−0.021, −0.008−0.001, 0.004	*NS* *NS* *NS* *NS* *NS* *NS* *NS* *NS* *NS* *NS* *NS* *NS* *NS*	−0.032, 0.038−1.8e^**−**6^, 0.008−0.006, 0.004−0.014, 0.001−0.006, 0.021−0.015, 0.003−0.008, 0.008−0.007, 0.003−0.007, 0.004−0.015, 0.02−0.013, 0.066−0.01, 0.005−0.001, 0.004
**tACl** **C14** **C14:1** **C14:2** **C14**–**OH** **C14:1**–**OH** **C16** **C16:1** **C16**–**OH** **C16:1**–**OH** **C18** **C18:1** **C18:2** **C18**–**OH** **C18:1**–**OH** **C18:2**–**OH**	**4.558**0.2770.1320.0810.0350.0412.4540.1620.0370.0561.4681.9380.3500.0330.0960.214	1.79–18.080.03–0.7330.03–0.6280–0.7080–0.230–0.2510.87–9.9490–0.4550–0.1690–0.3300.44–6.8570.61–9.2130.07–1.7160–0.1750.004–0.310.02–0.808	**5.223**0.2700.1270.0600.0300, 0403.0180.1680.0420.0551.5911.9040.2440.0360.0960.221	1.13–19.510.04–1.1380–0.8130–1.1510–0.2590–0.3770.29–12.7870–0.8510–0.3420–0.3460.29–5.7440.3–6.9530.02–1.2630–0.2830–0.5020.002–0.956	**5.503**0.2770.1250.0600.0360.0443.2520.1830.0430.0541.6301.9160.2340.0360.0900.211	2.22–16.020.09–1.050–0.9760–0.7540–0.1540–0.4071.06–10.7930.01–0.7950–0.2240–0.3160.368–4.9320.738–5.0510.041–1.1050–0.1580.007–0.3950–0.932	**4.01e** ^**−7**^ *NS* *NS* **2.5e** ^**−2**^ *NS* *NS* **2.1e** ^**12**^ *NS* *NS* *NS* *NS* *NS* **6.8e** ^**−17**^ *NS* *NS* *NS*	–0.837, −0.446−0.01, 0.017−0.004, 0.0130.012, 0.026−0.001, 0.004−0.004, 0.003−0.655, −0.402−0.016, 0.004−0.006, 0.001−0.002, 0.005−0.181, 0.002−0.002, 0.1260.085, 0.12−0.005, 0.004−0.011, 0.004−0.028, 0.001	*NS* *NS* *NS* *NS* *NS* *NS* *NS* *NS* *NS* *NS* *NS* *NS* *NS* *NS* *NS* *NS*	0.06, 0.476−0.004, 0.024−0.01, 0.006−0.004, 0.008−0.001, 0.005−0.001, 0.0060.09, 0.367−0.004, 0.026−0.003, 0.003−0.004, 0.004−0.044, 0.116−0.026, 0.131−0.02, 0.012−0.002, 0.003−0.009, 0.005−0.031, 0.008
**tAC/FC**	**0.657**	0.31–1.651	**0.728**	0.246–2.42	**0.795**	0.332–1.634	**0.005**	–0.101, −0.03	**0.036**	0.019, 0.094
**FC/TC**	**0.603**	0.37–0.761	**0.578**	0.292–0.803	**0.557**	0.38–0.751	**0.014**	0.011, 0.033	**0.0001**	−0.031, −0.009

p^1^, comparison between SGA and AGA newborns; p^2^, comparison between AGA and LGA newborns; 95% CI, 95% confidence interval; FC, free carnitine; TC, total carnitine; tAC, total acylcarnitines; tACs, total short-chain acylcarnitines; tACm, total medium-chain acylcarnitines; tACl, total long-chain acylcarnitines; SGA, small for gestational age; AGA, appropriate for gestational age; LGA, large for gestational age; NS, p not significant.

.

Although severe LGA newborns had higher levels of FC, TC, tAC, tACs, tACm, and tACl, the only significant difference compared with AGA^(3-97)^ newborns were higher levels of C3 (*p* < 0.05) (Table 2

**Table 2:** Acylcarnitine profiles (µmol/L) according to birth weight based on 3^rd^ and 97^th^ percentiles.

	SGA (n:75)		AGA^(3–97)^ (n:2364)		LGA (n:75)		SGA VS AGA		AGA VS LGA	
	Median	Range	Median	Range	Median	Range	*P* ^*1*^	*95%CI*	*P* ^*2*^	*95% CI*
**FC**	35.43	14.22–64.09	28.31	7.12–100.56	28.22	11.58–73.43	**0.009**	4.01,9.29	*NS*	−2.29, 2.685
**TC**	62.31	25.14–105.97	50.12	17.00–135.89	52.33	25.14–114.70	**1.72e** ^**−6**^	8.046, 16.168	*NS*	−2.361, 5.609
**tAC**	25.29	10.92–55.98	21.07	7.95–69.41	23.07	13.16–49.65	**0.0001**	2.607, 5.995	*NS*	0.131, 3.203
**tACs** **C2** **C3** **C3:1** **C4** **C4**–**OH** **C5** **C5:1** **C5**–**OH**	**19.41**14.603.670.0020.4430.3370.4890.0320.175	8.02–50.205.84–43.781.00–10.060–0.1280.03–1.530–0.9110.128–1.0480–0.1890.007–0.431	**14.51**11.672.220.0080.3020.2660.1790.0260.151	5.19–62.234.06–58.850.461–9.830–1.750–1.6770–1.9630–1.5780–0.8060–0.888	**16.24**12.952.8530.0110.2690.2930.1610.0280.167	8.23–41.435.35–36.480.93–5.620–0.1540.10–1.340.045–0.9780.018–1.090–0.1080.032–0.713	**9.16e** ^**−9**^ **0.012** **4.61e** ^**−8**^ *NS* **9.66e** ^**−5**^ *NS* **1.30e** ^**−15**^ *NS* *NS*	3.472, 6.3571.651, 3.9811.034, 1.732−3.7e^**−**5^, 1.8e^**−**5^0.094, 0.1790.034, 0.0970.239, 0.3496.3e^**−**6^, 0.0110.004, 0.034	*NS* *NS* **0**, **015** *NS* *NS* *NS* *NS* *NS* *NS*	0.278, 2.7450.171, 2.120.228, 0.76−4.8e^**−**5^, 2.3e^**−**5^−0.049, 0.011−0.019, 0.044−0.023, 0.013−0.003, 0.0060.0002, 0.031
**tACm** **C6** **C6**–**OH** **C8** **C8:1** **C10** **C10:1** **C10:2** **C5DC** **C4DC** **C12** **C12:1** **C12:2**	**0.688**0.060.0510.1190.1630.1130.0970.0450.080.2210.1250.0730.025	0.171–1.4710–0.2680–0.1930–0.3610–0.5940.006–0.3140–0.2510–0.1670–0.3320.04–0.5960–0.3870.006–0.1810–0.114	**0.762**0.050.0570.1160.1950.1260.1020.0520.0680.3290.1520.0920.026	0.109–7.0570–0.7310–0.4790–2.3940–0.6830–0.9880–0.790–0.4140–0.3950–1.0630–4.3910–0.5020–0.277	**0.779**0.0520.0630.1230.2180.1330.1100.0670.070.3250.1570.090.029	0.376–3.1080–0.3490–0.4030.026–0.460.039–0.4530–0.2840–0.2520–0.230–0.1780.149–1.1060.047–1.5540–0.4610–0.115	*NS* *NS* *NS* *NS* *NS* *NS* *NS* *NS* *NS* **4.86e** ^**−8**^ **4.79e** ^**−6**^ **0.0007** *NS*	−0.121, 0.003−6.7e^**−**5^, 0.025−0.013, 0.005−0.008, 0.018−0.043, 0.005−0.03, −0.001−0.02, 0.011−0.015, 0.004−0.001, 0.021−0.12, −0.068−0.062, −0.03−0.031, −0.012−0.003, 0.007	*NS* *NS* *NS* *NS* *NS* *NS* *NS* *NS* *NS* *NS* *NS* *NS* *NS*	−0.028, 0.087−0.005, 0.013−0.005, 0.013−0.006, 0.02−0.01, 0.034−0.017, 0.013−0.017, 0.0145.1e^**−**6^, 0.022)−0.007, 0.013−0.033, 0.026−0.021, 0.092−0.014, 0.016−2.8e^**−**5^, 0.011
**tACl** **C14** **C14:1** **C14:2** **C14**–**OH** **C14:1**–**OH** **C16** **C16:1** **C16**–**OH** **C16:1**–**OH** **C18** **C18:1** **C18:2** **C18**–**OH** **C18:1**–**OH** **C18:2**–**OH**	**4.47**0.2610.1330.0810.030.0382.240.1670.0340.0551.4502.020.3780.0280.0760.225	2.239–7.0950.047–0.6580.038–0.460–0.5920–0.1050–0.1320.871–4.3280–0.4410–0.1690–0.1620.583–2.7510.989–3.70.089–0.9910–0.1130.014–0.2740.039–0.797	**5.54**0.2720.1280.0610.0350.0422.9960.1690.0420.0551.5801.900.2500.0370.0960.219	1.139–19.510.037–1.1380–0.9760–1.1510–0.2590–0.4070.294–12.780–0.8510–0.3420–0.3460.29–6.8570.3–9.2130.022–1.7160–0.2830–0.5020–0.956	**5.98**0.2660.1270.0690.0380.0473.300.1760.0440.0551.671.900.2660.0390.1010.240	3.045–11.3260.126–0.9770–0.9390–0.7290–0.2590–0.1841.655–6.5290.05–0.7950–0.1670.009–0.3160.518–3.0380.987–3.8090.041–0.5670–0.1580.019–0.2240.027–0.831	**2.86e** ^**−8**^ *NS* *NS* *NS* *NS* *NS* **9.39e** ^**−12**^ *NS* *NS* *NS* *NS* *NS* **1.31e** ^**−6**^ **0.016** *NS* *NS*	−1.13, −0.482−0.02, 0.0190.01, 0.020.008, 0.03−0.008, 0.002−0.01, −0.001−0.95, −0.524−0.025, 0.011−0.01, −0.002−0.005, 0.007−0.177, 0.033−0.073, 0.210.093, 0.16−0.013, −0.004−0.02, −4.3e^**−**4^−0.04, 0.018	*NS* *NS* *NS* *NS* *NS* *NS* *NS* *NS* *NS* *NS* *NS* *NS* *NS* *NS* *NS* *NS*	−0.046, 0.648−0.022, 0.028−0.015, 0.015−0.007, 0.017−0.004, 0.0070.001, 0.0130.007, 0.459−0.007, 0.031−0.004, 0.006−0.006, 0.006−0.078, 0.148−0.054, 0.225−0.023, 0.042−0.001, 0.008−0.005, 0.02−0.031, 0.039
**tAC/FC**	**0.693**	0.314–1.58	**0.724**	0.246–2.42	**0.776**	0.431–1.634	*NS*	−0.096, 0.042	*NS*	−0.014, 0.112
**FC/TC**	**0.591**	0.388–0.761	**0.580**	0.292–0.803	**0.563**	0.38–0.699	*NS*	−0.008, 0.032	*NS*	−0.038, 0.001

p^1^, comparison between severe SGA and AGA^(3–97)^ groups; p^2^, comparison between AGA^(3–97)^ and severe LGA groups; 95% CI, 95% confidence interval; FC, free carnitine; TC, total carnitine; tAC, total acylcarnitines; tACs, total short-chain acylcarnitines; tACm, total medium-chain acylcarnitines; tACl, total long-chain acylcarnitines; SGA, small for gestational age; AGA, appropriate for gestational age; LGA, large for gestational age; NS, p not significant.

). In addition, no increase in carnitine deficiency or insufficiency markers was observed in this group.

The comparison between LGA-GDM and LGA-noGDM newborns revealed that a history of gestational diabetes was associated with higher levels of FC, TC, and short-chain acylcarnitines, including propionylcarnitine (LGA-GDM: 3.33 µmol/L; LGA-noGDM: 2.69 µmol/L), and lower levels of medium- and long-chain acylcarnitines, although the differences were not significant (Table 3). Analysis of the individual acylcarnitines revealed no remarkable differences between the two subgroups.

**Table 3:** Acylcarnitine pattern (µmol/L) in LGA newborns according to maternal history of gestational diabetes.

	LGA-noGDM (n:204)		LGA-GDM (n:42)		*P*	95% CI
	Median	Range	Median	Range		
FC	27.93	9.235–80.164	30.17	19.11–58.364	*NS*	−5.81, 0.93
TC	50.48	23.265–114.703	54.17	37.47–103.696	*NS*	−9.16, 1.13
tAC	22.29	11.831–49.653	23.37	17.241–45.332	*NS*	−3.88, 0.64
tACs	15.65	7.474–41.438	17.11	11.224–36.76	*NS*	−3.69, −0.05
tACm	0.77	0.202–7.057	0.807	0.483–1.557	*NS*	−0.10, 0.06
tACl	5,56	2.228–16.023	5.081	2.879–9.113	*NS*	−0.16, 0.88
tAC/FC	0.809	0.332–1.634	0.777	0.505–1.624	*NS*	−0.09, 0.11
FC/TC	0.553	0.38–0.751	0.563	0.381–0.664	*NS*	−0.03, 0.02

GDM, gestational diabetes mellitus; FC, free carnitine; TC, total carnitine; tAC, total acylcarnitines; tACs, total short-chain acylcarnitines; tACm, total medium-chain acylcarnitines; tACl, total long-chain acylcarnitines; SGA, small for gestational age; AGA, appropriate for gestational age; LGA, large for gestational age; NS, p not significant; 95% CI, 95% confidence interval.

## Discussion

Disturbances in fetal nutrition, such as intrauterine growth restriction (IUGR) and macrosomia, can impact health during adolescence and adulthood^13–16,34^ and are risk factors for later overweight^17–20,35,36^. Animal studies have shown that fetal overnutrition results in increased adiposity in newborns, leading to insulin resistance similar to that seen in cases of postnatal overnutrition^37^. Given the high worldwide prevalence of child obesity and overweight^38^, improved knowledge of metabolic homeostasis in higher-risk subgroups, such as LGA newborns, is essential for identifying possible treatment targets.

Recent investigations have identified abnormalities in the metabolic profiles of obese adults and children, such as increased plasma concentrations of branched-chain amino acids (BCAAs) (valine, leucine, and isoleucine), BCAA metabolism byproducts (e.g. alanine, glutamate/glutamine), C3 and C5 acylcarnitines, and aromatic amino acids (phenylalanine and tyrosine)^29,39–44^. The increase in BCAA and short-chain acylcarnitine concentrations is linked to elevated protein intake^45^, and levels are positively correlated with adiposity^46,47^ and strongly associated with IR^29,32,44,48^. The metabolic environment in LGA is less well-known. Higher levels of adipokines^49^, BCAAs, and other metabolites, such as alanine, glutamine, threonine, citric acid, glycerol, and glucose, have been detected in cord-blood samples of LGA newborns^50^, and similar findings have been reported for myo-inositol levels in urine samples^51^.

Ours is the first study to characterize acylcarnitine profiles in LGA newborns. In line with the results of obesity studies in children^41,43^ and adults^29,31^, our data show that LGA newborns have increased postnatal levels of C3, a product of mitochondrial BCAA catabolism, and in particular of isoleucine and valine catabolism^29^. BCAAs act as signaling molecules of nutritional status^52^. Increased concentrations in obese and insulin-resistant humans may be caused by down-regulation of BCAA oxidation enzymes in adipose tissue^53,54^, as has been observed in animal models with genetic or diet-induced obesity^55^. Propionylcarnitine is a carnitine conjugate of propionyl-CoA, which has been identified as a potential substrate for odd-chain fatty acid synthesis^56^. Moreover, C3 and C5 levels are promising biomarkers for discriminating metabolic wellness in obese individuals^57^, as higher levels have been observed in metabolically unhealthy individuals, independently of body mass index^58^. Consistent with these observations, the LGA newborns in our study had higher C3 (*p* < 0.001) and C5 levels than AGA newborns, and the concentrations of C3 were even higher than those observed in obese children^41,43^ and adults^29^. This could reflect an unhealthy metabolic status in LGA newborns with potential complications in later life. The LGA group also had higher C2 plasma levels, and these were particularly evident in severe LGA newborns. C2 may play a role in insulin resistance through its interaction with acetyl-CoA via carnitine acetyl-CoA transferase. This enzyme seems to act as a positive regulator of total body glucose tolerance and muscle activity of pyruvate dehydrogenase^59^. C2 levels are increased in prediabetic states^60^, diabetes mellitus^30,32,60^, and metabolic syndrome^32^, and are significantly and positively correlated with HbA_1c_ levels^30^. Interestingly, the levels in the LGA group were higher than those reported for diabetic adult patients^30,32,60^.

One of the most notable differences between LGA and AGA newborns in our study was the significantly higher tACs levels in the LGA group (*p* < 0.0001). This observation is consistent with reports of increased tACs in obesity, impaired glucose tolerance, and diabetes mellitus^61^.

The acylcarnitine pattern of increased tAC, tACs, C2, and C3 levels was more evident in the SGA group, supporting previous observations in animal models^62^ and neonatal studies^63^, and suggesting an impaired fatty acid metabolism in both fetal growth disorders.

In agreement with the profile described for overweight adults^29^ and children^64^, LGA newborns also showed higher (though not significantly so) concentrations of medium- and long-chain acylcarnitines than AGA newborns.

A strong correlation was recently demonstrated between carnitine and body composition^65^. Although we observed slightly higher plasma concentrations of free carnitine in LGA newborns, our data also showed a greater tendency towards carnitine deficiency (*p* < 0.05) and insufficiency (*p* < 0.001) in this group. Increases in tAC/FC ratio precede a decrease in total plasma carnitine and indicate low tissue bioavailability of FC^66^. The higher FC levels in LGA contrast with the carnitine depletion reported for diet-induced obesity^67^. Nevertheless, metabolomic studies of obesity have also shown higher levels of carnitine in obese children^43^. Carnitine insufficiency in our cohort appears to be unrelated to antenatal exposure to GDM. This is relevant given the proposed causative role of carnitine insufficiency in mitochondrial dysfunction and obesity-related impairments in glucose tolerance^68^. It is also consistent with reports that document that GDM in pregnant women does not negatively affect the efficiency of the carnitine system^69^. We did not find any postnatal differences between acylcarnitine profiles in LGA-GDM and LGA-noGDM newborns in our study, although the former had higher concentrations of FC, TC, tAC and tACs. In line with this observation, higher FC and TC levels have been reported in pregnant women with GDM versus healthy pregnant women at 30–33 weeks of gestation^69^. We do not consider that the absence of significant differences between the LGA-GMD and LGA-no GDM groups is due to sample size, as the minimum detectable effect sizes for the samples used in each comparison (with 5% significance and 80% statistical power) were 0.2 for AGA vs LGA and AGA vs SGA (Table 1), 0.25 for SGA vs LGA (Table 1), 0.33 for AGA vs LGA (Table 2), and 0.48 for LGA-noGMD vs LGA-GMD (Table 3). This means that, even in the worst-case scenario (Table 3), we are able to detect true between-group differences of higher than 50% of the SD, which are considered medium effect sizes^70^.

Our findings describe a postnatal acylcarnitine profile in LGA newborns that is characteristic of obesity and associated with the development of insulin resistance and prediabetic states, supporting the view that early imbalance in metabolic homeostasis in LGA newborns could contribute to deleterious effects in the long term. Identification of this profile, linked to an unhealthy metabolic phenotype, in the postnatal period could help to establish early dietary intervention and follow-up to reduce the risk of overweight and metabolic syndrome in later life.

## Patients and Methods

### Study design

The acylcarnitine profiles of LGA newborns were determined in a 1-year observational study approved by the Research Ethics Committee of Galicia, Spain (registry number 2015/315). The processing of clinical data for research purposes at the beginning of the study and the study protocol complied with the principles of the Helsinki Declaration of 1964, as revised in October 2013 in Fortaleza, Brazil.

### Patients

This study was conducted at Hospital Clínico Universitario de Santiago de Compostela, a tertiary hospital in north-west Spain. All newborns born in or referred to our hospital during the first 48 hours of life over the period of 1 year (2015) were included in the study. Informed consent was obtained from parents or legal guardians. Infants with an established diagnosis of an inborn error of metabolism known to alter acylcarnitine profiles (primary systemic carnitine deficiency, mitochondrial fatty acid ß-oxidation defects, ß-ketothiolase deficiency, propionic, methylmalonic or isovaleric acidemia, 3-methylcrotonyl-CoA carboxylase deficiency, 3-hydroxy-3-methylglutaryl-CoA lyase deficiency, or 3-methylglutaconic aciduria) were excluded from the analysis. The following variables were recorded at birth: sex, gestational age, weight (g), length (cm), head circumference (cm), history of GDM, and treatments received (dietary and/or insulin treatment).

Newborns were classified into the following groups according to their birth weight: AGA (≥10^th^ percentile and ≤90^th^ percentile of birth weight for GA), SGA (<10^th^ percentile of birth weight for GA), and LGA (>90^th^ percentile of birth weight for GA). For additional analyses we also classified newborns with a birth weight greater than the 97^th^ percentile for GA into a severe LGA group.

Birth weight percentiles and z-scores for GA were calculated using the online nutritional assessment tool of the Spanish Society of Gastroenterology, Hepatology and Nutrition (www.gastroinf.es), which is based on Spanish neonatal growth curves^71^.

GDM was defined according to the criteria established in the 2016 Guidelines of the American Diabetes Association^72^ using the two-step diagnostic strategy: 1) if plasma glucose is ≥140 mg/dL (7.8 mmol/L) in the 1-hour glucose loading-test, pregnant women must 2) undergo a glucose tolerance test (administration of 100 g of glucose after 8 hours of fasting with subsequent sequential blood sampling). Diagnosis is confirmed when two or more of the following glucose criteria are fulfilled: fasting, ≥105 mg/dL (5.8 mmol/L); 1 hour, ≥190 mg/dL (10.6 mmol/L); 2 hours, ≥165 mg/dL (9.2 mmol/L); and 3 hours, ≥145 mg/dL (8.0 mmol/L).

Based on the corresponding obstetric history of gestational diabetes, LGA newborns were sub-classified as LGA-GDM (co-occurrence of gestational diabetes) or LGA-noGDM (absence of GDM).

### Tandem mass spectrometry and study parameters

Analyses of free carnitine and acylcarnitines were performed on a tandem mass spectrometer coupled to a triple quadrupole analyzer (ESI-MS/MS API 2000; Applied Biosystems Sciex, Toronto, Canada) following an established methodology^73^.

The plates were prepared using the following protocol. The paper blood sample disks and patterns were cut using a BSD 700 automatic drill (BSD tech., Brisbane, Australia) and hand drills followed by microplate placement and addition to each well of methanol. Acylcarnitines were purified with methanol and stable isotope-labeled patterns were used to determine their respective concentrations. The acylcarnitines were then extracted by vortex shaking for 25 minutes. Subsequently, all the methanol was transferred to another plate to distil the blood disks and then evaporated in a gas extractor. The acylcarnitines were derivatized with butanol to their butyl-esters in an acid medium to increase the selectivity of the technique. This was done with the addition of 3N HCl in n-butanol followed by heating at 65 ± 5 °C for 20 minutes and cooling for 5 minutes in a freezer. Excess butanol was evaporated to dryness and once the evaporated plates were at room temperature, a new solution was prepared with 100 μL of the mobile phase of the chromatograph (acetonitrile: water, 1:1). The plates were then covered with foil, vortexed for 5 minutes, and finally analyzed (precursor m/z 120–280 amu).

The reagents were prepared using water purified with a Milli-Q system (Millipore) and the mobile phase was composed of acetonitrile (LiCrosolv Merck, ref. 00030) and formic acid 0.005% (Merck, ref.02264).

A comprehensive analysis of acylcarnitine profiles was conducted by MS/MS using dried-blood spots collected on the third day of life for expanded newborn screening. We analyzed: *short-chain acylcarnitines*: acetyl- (C2), propionyl- (C3), propenyl- (C3:1), C4-, 3-OH-butyryl- (C4-OH), C5- and tiglyl-carnitine (C5:1); *medium-chain acylcarnitines*: C6-, 3-hydroxy-hexanoyl- (C6-OH), C8-, octenoyl- (C8:1), methylmalonyl- (C4DC), C10-, decenoyl- (C10:1), decadienoyl- (C10:2), C12- and dodecenoyl-carnitine (C12:1); and *long-chain acylcarnitines*: C14-, myristoleyl- (C14:1), hydroxymyristoyl- (C14-OH), C16-, hexadecenoyl- (C16:1), 3-hydroxi-hexadecanoyl- (C16-OH), 3-hydroxypalmitoleylc- (C16:1-OH), C18-, oleyl- (C18:1), linoleyl- (C18:2), hydroxyoleyl- (C18:1-OH) and 3-hydroxy-linoleyl-carnitine (C18:2-OH). It should be noted that the analytical method employed does not allow for the differentiation of isobaric acylcarnitines. The following parameters were also assessed: total short-chain acylcarnitines (tACs), total medium-chain acylcarnitines (tACm), total long-chain (tACl) acylcarnitines (the sum of short-, medium-, and long-chain acylcarnitines, respectively), total acylcarnitines (tAC) (the sum of all acylcarnitines studied); total carnitine (TC), defined as the sum of free carnitine (FC) and total acylcarnitines (tAC), FC/TC ratio (values <0.54 in neonates are suggestive of carnitine deficiency), and tAC/FC ratio (values >0.83 in newborns are indicative of carnitine insufficiency)^74^. We also evaluated three acylcarnitine ratios typically included in neonatal screening: C8/C2, C8/C10, and FC/C16.

### Statistical analyses

Data were analyzed using the R statistical package (version 3.2.1; R Project for Statistical Computing). Sample normality was assessed using the Kolmogorov-Smirnov test. ANOVA was used to compare normally distributed data, and the Kruskal–Wallis test was used to compare non-normally distributed data. Qualitative variables were compared using Fisher’s exact test. Normal samples with unknown variance were compared using Student’s t-test, while non-normally distributed data were compared using the Wilcoxon rank test. Finally, the *p*-values obtained were adjusted using Bonferroni correction. Only adjusted *p*-values < 0.05 were considered statistically significant.

## Electronic supplementary material

supplementary information

## References

[1] Das UG, Sysyn GD (2004). Abnormal fetal growth: intrauterine growth retardation, small for gestational age, large for gestational age. Pediatr. Clin. N. Am..

[2] Perlow JH, Morgan MA, Montgomery D, Towers CV, Porto M (1992). Perinatal outcome in pregnancy complicated by massive obesity. Am. J. Obstet. Gynecol..

[3] Vohr BR, McGarvey ST, Coll CG (1995). Effects of maternal gestational diabetes and adiposity on neonatal adiposity and blood pressure. Diabetes Care.

[4] Djelantik AA, Kunst AE, van der Wal MF, Smit HA, Vrijkotte TG (2012). Contribution of overweight and obesity to the occurrence of adverse pregnancy outcomes in a multi-ethnic cohort: population attributive fractions for Amsterdam. BJOG.

[5] Oyarzo C (2012). Adverse perinatal outcomes after the February 27th 2010 Chilean earthquake. J. Matern. Fetal Neonatal Med..

[6] Blackwell SC (2016). Relationship between excessive gestational weight gain and neonatal adiposity in women with mild gestational diabetes mellitus. Obstet. Gynecol..

[7] Wang LF (2015). Influence of pre-pregnancy obesity on the development of macrosomia and large for gestational age in women with or without gestational diabetes mellitus in Chinese population. J. Perinatol..

[8] Su R (2016). Relationship of maternal birth weight on maternal and neonatal outcomes: a multicenter study in Beijing. J. Perinatol..

[9] Zilberlicht A (2016). The mutual effect of pregestational body mass index, maternal hyperglycemia and gestational weight gain on adverse pregnancy outcomes. Gynecol. Endocrinol..

[10] Ruiz M (2015). Mother’s education and the risk of preterm and small for gestational age birth: a DRIVERS meta-analysis of 12 European cohorts. J. Epidemiol. Community Health.

[11] Sridhar SB, Ferrara A, Ehrlich SF, Brown SD, Hedderson MM (2013). Risk of large-for-gestational-age newborns in women with gestational diabetes by race and ethnicity and body mass index categories. Obstet. Gynecol..

[12] Tutlam, N. T., Liu, Y., Nelson, E. J., Flick, L. H. & Chang, J. J. The effects of race and ethnicity on the risk of large-for-gestational-age newborns in women without gestational diabetes by prepregnancy body mass index categories. *Matern. Child Health*10.1007/s10995-016-2256-x (2017). 10.1007/s10995-016-2256-x 28092059

[13] Herva A (2008). Birth measures and depression at age 31 years: the Northern Finland 1966 Birth Cohort Study. Psychiatry Research.

[14] Fabricius-Bjerre S (2011). Impact of birth weight and early infant weight gain on insulin resistance and associated cardiovascular risk factors in adolescence. PLoS One.

[15] Zhang Y (2013). The associations of high birth weight with blood pressure and hypertension in later life: a systematic review and meta-analysis. Hypertens. Res..

[16] Kuciene, R., Dulskiene, V. & Medzioniene, J. Associations between high birth weight, being large for gestational age, and high blood pressure among adolescents: a cross-sectional study. *Eur. J. Nutr*, 10.1007/s00394-016-1372-0 (2017). 10.1007/s00394-016-1372-0 PMC5847040 28058464

[17] Hediger ML (1998). Muscularity and fatness of infants and young children born small- or large-for-gestational-age. Pediatrics.

[18] Eyzaguirre F (2012). Prevalence of components of the metabolic syndrome according to birthweight among overweight and obese children and adolescents. J. Pediatr. Endocrinol. Metab..

[19] Sparano S (2013). Being macrosomic at birth is an independent predictor of overweight in children: results from the IDEFICS study. Matern. Child Health J..

[20] Taal HR, Vd Heijden AJ, Steegers E, Hofman A, Jaddoe V (2013). Small and large size for gestational age at birth, infant growth, and childhood overweight. Obesity.

[21] Boney CM, Verma A, Tucker R, Vohr BR (2005). Metabolic syndrome in childhood: association with birth weight, maternal obesity, and gestational diabetes mellitus. Pediatrics.

[22] Wang X, Liang L, Junfen FU, Lizhong DU (2007). Metabolic syndrome in obese children born large for gestational age. Indian J. Pediatr..

[23] Giapros V (2007). Serum metabadiponectin and leptin levels and insulin resistance in children born large for gestational age are affected by the degree of overweight. Clin. Endocrinol. (Oxf).

[24] Cetin C (2014). Comparative analysis of glucoinsulinemic markers and proinflammatory cytokines in prepubertal children born large-versus appropriate-for gestational age. Endocrine.

[25] Giapros V, Cholevas VI, Evagelidou EN, Bairaktari ET, Andronikou SK (2014). Vitamin D, parathormone and insulin resistance in children born large for gestational age. J. Pediatr. Endocrinol. Metab..

[26] Xie C, Wang Y, Li X, Wen X (2016). Childhood growth trajectories of etiological subgroups of large for gestational age newborns. J. Pediatr..

[27] Arner P (2005). Human fat cell lipolysis: biochemistry, regulation and clinical role. Best Pract. Res. Clin. Endocrinol. Metab..

[28] Goossens GH (2008). The role of adipose tissue dysfunction in the pathogenesis of obesity-related insulin resistance. Physiol. Behav..

[29] Newgard CB (2009). A branched-chain amino acid-related metabolic signature that differentiates obese and lean humans and contributes to insulin resistance. Cell. Metab..

[30] Adams SH (2009). Plasma acylcarnitine profiles suggest incomplete long-chain fatty acid β-oxidation and altered tricarboxylic acid cycle activity in type 2 diabetic African-American women. J. Nutr..

[31] Mihalik SH (2010). Increased levels of plasma acylcarnitines in obesity and type 2 diabetes and identification of a marker of glucolipotoxicity. Obesity.

[32] Bene J (2013). Similarities in serum acylcarnitine patterns in type 1 and type 2 diabetes mellitus and in metabolic syndrome. Ann. Nutr. Metab..

[33] Huynh J, Xiong G, Bentley-Lewis R (2014). A systematic review of metabolite profiling in gestational diabetes mellitus. Diabetologia.

[34] Longo S (2013). Short-term and long-term sequelae in intrauterine growth retardation (IUGR). J. Matern. Fetal Neonatal Med..

[35] Yu ZB (2011). Birth weight and subsequent risk of obesity: a systematic review and meta-analysis. Obes. Rev..

[36] Schellong K, Schulz S, Harder T, Plagemann A (2012). Birth weight and long-term overweight risk: systematic review and a meta-analysis including 643,902 persons from 66 studies and 26 countries globally. PLoS One.

[37] Liu Z (2013). Neonatal overnutrition in mice exacerbates high-fat diet-induced metabolic perturbations. J. Endocrinol..

[38] Ng M (2014). Global, regional, and national prevalence of overweight and obesity in children and adults during 1980-2013: a systematic analysis for the Global Burden of Disease Study 3013. Lancet.

[39] Jourdan C (2012). Body fat free mass is associated with the serum metabolite profile in a population-based study. PLoS One.

[40] McCormack SE (2013). Circulating branched-chain amino acid concentrations are associated with obesity and future insulin resistance in children and adolescents. Pediatr. Obes..

[41] Perng W (2014). Metabolomic profiles and childhood obesity. Obesity.

[42] Moore SC (2014). Human metabolic correlates of body mass index. Metabolomics.

[43] Butte NF (2015). Global metabolomic profiling targeting childhood obesity in the Hispanic population. Am. J. Clin. Nutr..

[44] Zhao X (2016). Using metabolomic profiles as biomarkers for insulin resistance in childhood obesity: a systematic review. J. Diabetes Res..

[45] Hellmuth C (2016). Effects of early nutrition on the infant metabolome. Nestle Nutr. Inst. Workshop Ser..

[46] Boulet MM (2015). Alterations of plasma metabolite profiles related to adipose tissue distribution and cardiometabolic risk. Am. J. Physiol. Endocrinol. Metab..

[47] Rietman A (2016). Associations between plasma branched-chain amino acids, β-aminoisobutyric acid and body composition. J. Nutr. Sci..

[48] Zhao X (2016). The relationship between branched-chain amino acid related metabolomic signature and insulin resistance: a systematic review. J. Diabetes Res..

[49] Lausten-Thomsen U, Christiansen M, Hedley PL, Holm JC, Schmiegelow K (2016). Adipokines in umbilical cord blood from children born large for gestational age. J. Pediatr. Endocrinol. Metab..

[50] Fotakis C (2016). Investigating the metabolic fingerprint of term infants with normal and increased fetal growth. RSC Adv..

[51] Dessi A (2014). Investigation of the ^1^H-NMR based urine metabolomic profiles of IUGR, LGA and AGA newborns on the first day of life. J. Matern. Fetal Neonatal Med..

[52] Bifari, F. & Nisoli, E. Branched-chain amino acids differently modulate catabolic and anabolic states in mammals: a pharmacological point of view. *Br. J. Pharmacol*, 10.1111/bph.13624 (2016). 10.1111/bph.13624 PMC5429325 27638647

[53] Pietilaäinen KH (2008). Global transcript profiles of fat in monozygotic twins discordant for BMI: pathways behind acquired obesity. PLoS Med..

[54] Herman MA, She P, Peroni OD, Lynch CJ, Kahn B (2010). Adipose tissue branched chain amino acid (BCAA) metabolism modulates circulating BCAA levels. J. Biol. Chem..

[55] Estrada-Alcalde I (2017). Metabolic fate of branched-chain amino acids during adipogenesis, in adipocytes from obese mice and C2C12 myotubes. J. Cell. Biochem..

[56] Crown SB, Marze N, Antoniewicz MR (2015). Catabolism of branched chain amino acids contributes significantly to synthesis of odd-Chain and even-chain fatty acids in 3T3-L1 adipocytes. PLoS One.

[57] Gao X (2016). Serum metabolic biomarkers distinguish metabolically healthy peripherally obese from unhealthy centrally obese individuals. Nutr. Metab..

[58] Batch BC (2013). Branched chain amino acids are novel biomarkers for discrimination of metabolic wellness. Metabolism.

[59] Muoio DM (2012). Muscle-specific deletion of carnitine acetyltransferase compromises glucose tolerance and metabolic flexibility. Cell. Metab..

[60] Mai M (2013). Serum levels of acylcarnitines are altered in prediabetic conditions. PLoS One.

[61] Inokuchi T, Imamura K, Nomura K, Nomoto K, Isogai S (1995). Changes in carnitine metabolism with ketone body production in obese glucose-intolerant patients. Diabetes Res. Clin. Pract..

[62] Beauchamp B (2015). Undernutrition during pregnancy in mice leads to dysfunctional cardiac muscle respiration in adult offspring. Biosci. Rep..

[63] Liu J, Chen XX, Li XW, Fu W, Zhang WQ (2016). Metabolomic research on newborn infants with intrauterine growth restriction. Medicine (Baltimore)..

[64] Wahl S (2012). Childhood obesity is associated with changes in the serum metabolite profile. Obes. Facts.

[65] Murphy, R. A. *et al*. Metabolites associated with lean mass and adiposity in older black men. *J. Gerontol. A. Biol. Sci. Med. Sci*. 10.1093/gerona/glw245 (2017). 10.1093/gerona/glw245 PMC5861966 28052980

[66] Winter SC, Zorn EM, Vance WH (1990). Carnitine deficiency. Lancet.

[67] Schooneman MG (2016). The impact of altered carnitine availability on acylcarnitine metabolism, energy expenditure and glucose tolerance in diet-induced obese mice. Biochim. Biophys. Acta.

[68] Noland RC (2009). Carnitine insufficiency caused by aging and overnutrition compromises mitochondrial performance and metabolic controls. J. Biol. Chem..

[69] Pappa KI (2005). Gestational diabetes exhibits lack of carnitine deficiency despite relatively low carnitine levels and alterations in ketogenesis. J. Matern. Fetal Neonatal Med..

[70] Cohen, J. Statistical Power Analysis for the Behavioral Sciences, 2nd Edition. Lawrence Erlbaum, Hillsdale, New Jersey, USA, 1988).

[71] Carrascosa A (2008). Spanish cross-sectional growth study 2008. Part I: weight and height values in newborns of 26-42 weeks of gestational age. An. Pediatr..

[72] Diabetes Management Guidelines (2016). American Diabetes Association (ADA) 2016 Guidelines. Diabetes Care.

[73] Rinaldo P, Cowan TM, Matern D (2008). Acylcarnitine profile analysis. Genet. Med..

[74] Campoy C (1998). Evaluation of carnitine nutritional status in full-term newborn infants. Early Hum. Dev..

