# Supplementary material for: Similarities between acylcarnitine profiles in large for gestational age newborns and obesity
Source: Sci Rep. 2017 Nov 24;7:16267. doi: 10.1038/s41598-017-15809-4 (PMC5701125; doi:10.1038/s41598-017-15809-4)

Similarities between acylcarnitine profiles in large for gestational age newborns and obesity.

Paula Sánchez-Pintos, Maria-Jose de Castro MJ, Iria Roca, Segundo Rite, Miguel López Maria-Luz Couce.

Supplementary table 1.Maternal characteristics of the study cohort.

	All mothers (n:2514)	Mothers of LGA-GDM newborns (n:42)	Mothers of LGA-noGDM newborns (n:204)	*p*
Age (years)	32.82±4.31	34.69±4.53	32.79±5,39	*NS*
Steroid therapy	4.2%	0%	0,98%	*NS*
Maternal hypothyroidism	3.9%	11,11%	3.66%	*NS*
Preclampsia	2.1%	4,76%	0.99%	*NS*
Risk of preterm delivery	2.6%	0%	1.96%	*NS*

NS: not significant.

Supplementary Figure 1. Flow diagram of the cohort.

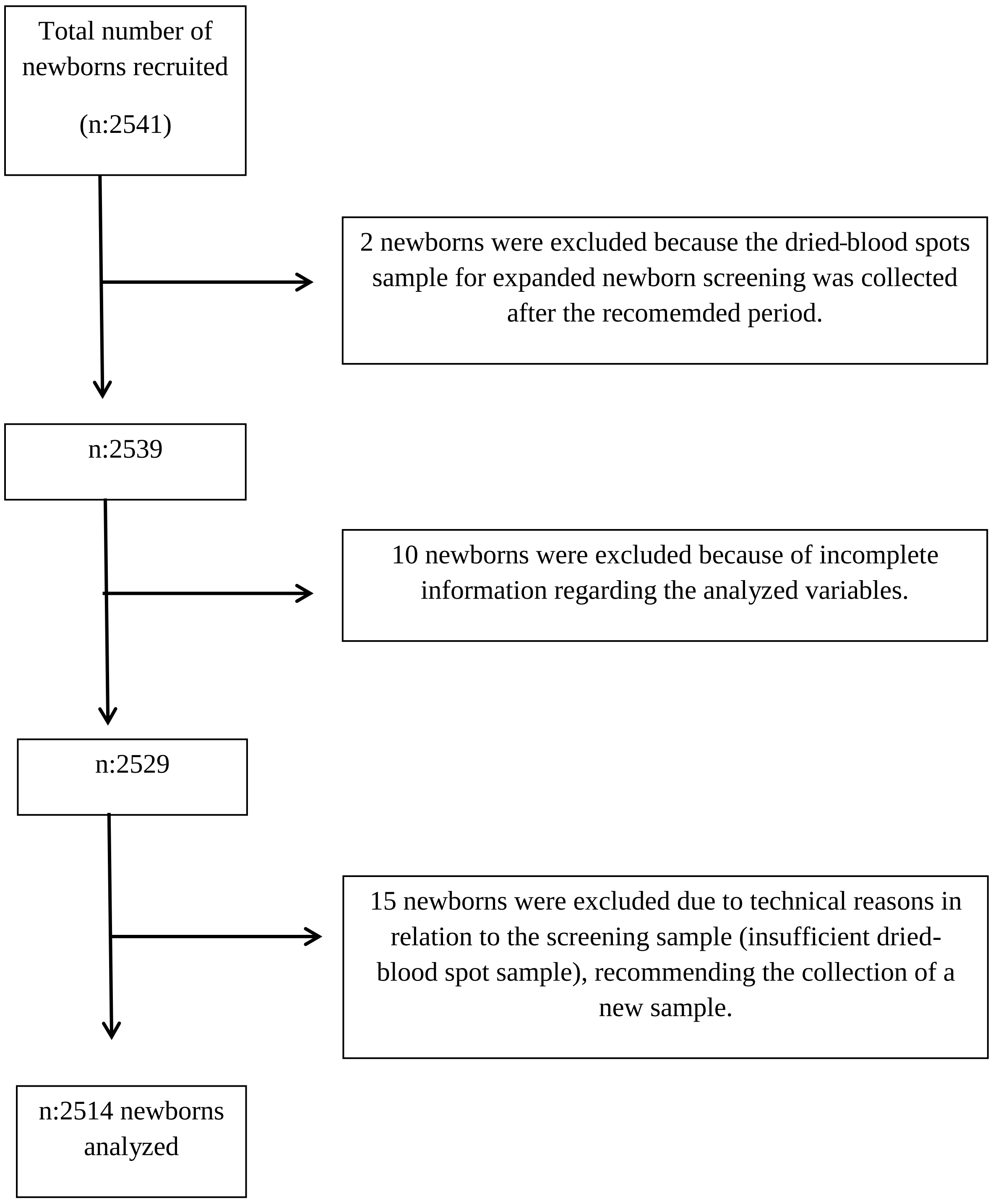

Supplement: Supplementary file 1 — supplementary information [file 41598_2017_15809_MOESM1_ESM.pdf]
